# Signet Ring Early Gastric Cancer: Seize the Opportunity

**DOI:** 10.1093/jcag/gwz049

**Published:** 2020-01-23

**Authors:** Robert Bechara, Haruhiro Inoue, David Hurlbut

**Affiliations:** 1 Division of Gastroenterology, Queens University, Kingston Health Sciences Center, Kingston, Ontario, Canada; 2 Digestive Diseases Center, Showa University, Koto-Toyosu Hospital Toyosu, Tokyo, Japan; 3 Department of Pathology and Molecular Medicine, Queens University, Kingston Health Sciences Center, Kingston, Ontario, Canada

**Figure 1.**
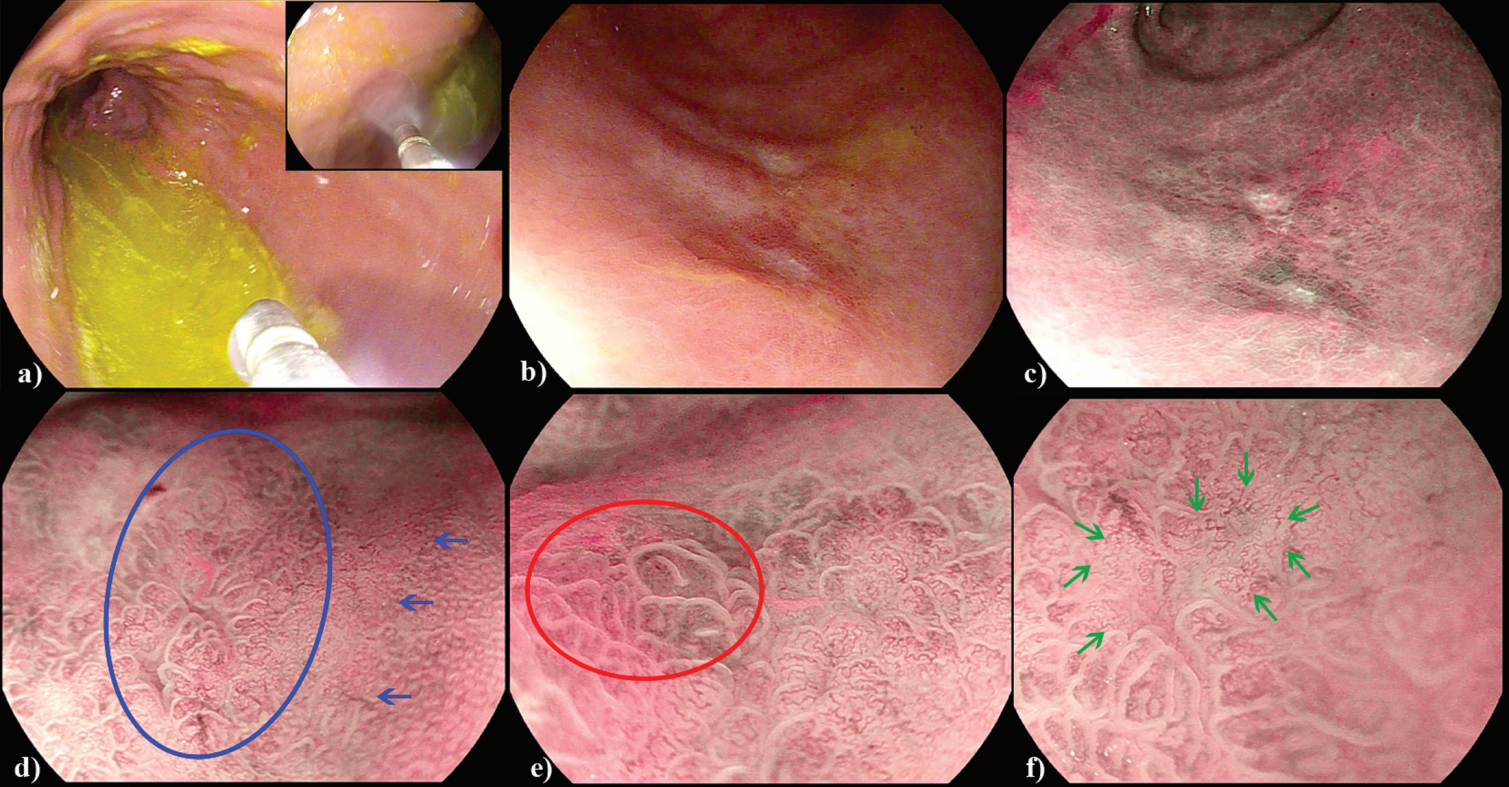
Early gastric cancer diagnosis. (a) bilious staining upon entry, washing with simethicone and mucolytic (b) lesion with white light (c) optical enhancement (d) magnification, blue arrows indicating demarcation between cancer (left) and normal mucosa (right) (e) stretch sign (red circle) due to infiltration by signet cells f) corkscrew pattern vessels (green arrows).

**Figure 2.**
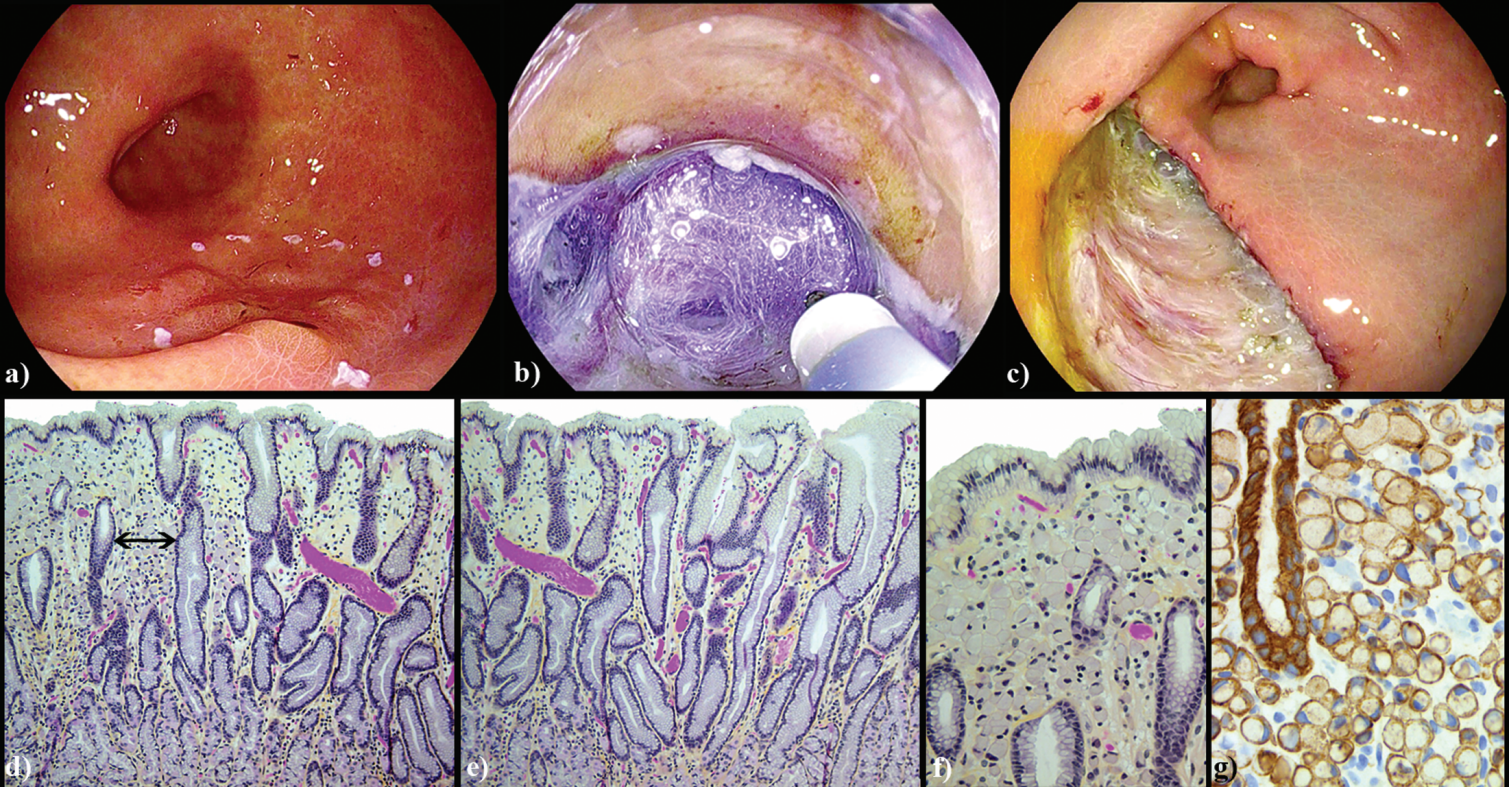
Early gastric cancer resection and pathology. (a–c) Marking and ESD of the lesion; (d) signet cell carcinoma; tumour cells infiltrate superficial lamina propria expanding inter-pit distance (black arrows), correlates with stretch sign (100× magnification) (e) normal inter-pit spacing of adjacent normal gastric mucosa (100× magnification) (f) signet ring carcinoma; tumour cells have cytoplasmic mucin vacuole and eccentrically displaced nucleus (200× magnification) (g) cytokeratin immunohistochemistry (400× magnification).

A 66-year-old Caucasian female presented for endoscopy due to nonspecific symptoms of abdominal pain and bloating. During endoscopy, random gastric and duodenal biopsies were taken due to reported ‘patchy erythema’ in the stomach and to rule out celiac, respectively. Surprisingly, a single gastric biopsy specimen demonstrated poorly differentiated signet ring gastric cancer. The procedure was repeated by an endoscopist trained in magnifying endoscopy. Upon entry into the stomach, there was copious bilious material and mucus (Figure 1a). After approximately 10 minutes of vigorous washing with simethicone and N-acetylcysteine, a thorough examination could be achieved (Figure 1b–d). The lesion was detected with white light endoscopy and demonstrated a Paris IIb+IIc morphology. The lesion was characterized with image-enhanced endoscopy and magnification (Figure 1b–f). It demonstrated findings suggestive of undifferentiated early gastric cancer (EGC), namely the stretch sign, corkscrew pattern vessels and intralobular loop pattern 2 (ILL-2) (Figure 1d–f) (1,2). A curative resection was achieved with Endoscopic Submucosal Dissection (ESD) (Figure 2) (3).

A misconception by Western endoscopists is that flat EGC do not occur in the western population. Although the incidence of gastric cancer in the west is relatively low, EGC do occur and can be missed (4). With every endoscopic exam, the opportunity to detect and potentially cure EGCs should be seized.
